# Clinical Performance of BIO-S and BIO-SC Composite Bioscores for 28-Day Mortality Stratification in Adults with Sepsis and Septic Shock

**DOI:** 10.3390/biomedicines14061271

**Published:** 2026-06-02

**Authors:** George Țocu, Bogdan Ioan Ștefănescu, Lavinia Țocu, Florentin Dimofte, Valerii Luțenco, Oana Mariana Mihailov, Raul Mihailov, Loredana Stavăr Matei

**Affiliations:** 1 Faculty of Medicine and Pharmacy, Research Center in the Medical-Pharmaceutical Field, “Dunărea de Jos” University, 800008 Galati, Romania; george.tocu@ugal.ro (G.Ț.); florentin.dimofte@ugal.ro (F.D.); valerii.lutenco@ugal.ro (V.L.); oana.mihailov@ugal.ro (O.M.M.); raul.mihailov@ugal.ro (R.M.); loredana.matei@ugal.ro (L.S.M.); 2 “Sf. Apostol Andrei” County Emergency Clinical Hospital, 800578 Galati, Romania; 3 “Sfântul Spiridon” Clinical Hospital of Pneumophthisiology, 800552 Galati, Romania; 4 “Sf. Ioan” Children’s Emergency Hospital, 800487 Galati, Romania

**Keywords:** sepsis, septic shock, composite bioscore, prognostic stratification, mortality prediction, risk assessment

## Abstract

**Background:** Short-term mortality stratification in sepsis remains clinically challenging, particularly because outcome is influenced by acute inflammation, coagulation abnormalities, organ dysfunction, and baseline comorbidity burden. This study evaluated the clinical performance of the BIO-S and BIO-SC composite bioscores for 28-day mortality stratification in adults with sepsis and septic shock. **Methods:** We conducted a prospective observational monocentric cohort study including 572 adult patients admitted between January 2022 and December 2024. BIO-S integrated procalcitonin (PCT), neutrophil-to-lymphocyte ratio (NLR), International Normalized Ratio (INR), and Sequential Organ Failure Assessment (SOFA) score, while BIO-SC extended this model by adding the Charlson Comorbidity Index (CCI). Prognostic performance was assessed using receiver operating characteristic (ROC) curve analysis, DeLong comparisons, bootstrap validation, calibration analysis, Kaplan–Meier survival curves, and Cox proportional hazards models. **Results:** The cohort included 418 patients with sepsis and 154 patients with septic shock. Overall 28-day mortality was 31.5% and was significantly higher in septic shock than in sepsis, 77.9% versus 14.4%, *p* < 0.001. BIO-S and BIO-SC showed strong discriminatory ability for 28-day mortality, with areas under the curve (AUCs) of 0.889 and 0.897, respectively. BIO-SC had the highest AUC, although the difference between BIO-SC and BIO-S was not statistically significant by the DeLong test, *p* = 0.328. At the optimal thresholds, BIO-S showed 97.8% sensitivity and 69.4% specificity, while BIO-SC showed 89.4% sensitivity and 77.8% specificity. Both bioscores stratified observed mortality across predefined risk categories and remained significantly associated with 28-day mortality in adjusted Cox models. **Conclusions:** BIO-S and BIO-SC showed clinically relevant performance for 28-day mortality stratification in adults with sepsis and septic shock. BIO-SC provided a numerically higher AUC and slightly better calibration, suggesting that comorbidity burden may improve prognostic characterization, although further independent multicenter validation is needed before broader clinical implementation.

## 1. Introduction

A substantial proportion of potentially preventable deaths in critically ill patients is still related to sepsis, which continues to represent a major challenge for modern medicine [1,2,3]. Early recognition of patients with an unfavorable prognosis remains difficult because clinical expression varies widely across individuals and is strongly influenced by comorbid conditions, infection-related severity, and differences in the host response to infection [4,5,6]. In routine clinical practice, prognostic assessment in sepsis often relies on the combined interpretation of clinical status, organ dysfunction, laboratory biomarkers, and the patient’s underlying vulnerability. However, the absence of sufficiently precise and easily applicable prognostic tools may limit early risk stratification and may negatively influence therapeutic prioritization, monitoring intensity, and patient outcome [7,8,9].

During the last two decades, a wide range of biomarkers has been investigated for severity assessment and outcome prediction in sepsis; however, no individual biomarker has demonstrated adequate stand-alone performance to function as a sufficiently reliable prognostic tool across heterogeneous adult sepsis populations [10,11]. Procalcitonin (PCT), C-reactive protein (CRP), interleukin-6 (IL-6), and the neutrophil-to-lymphocyte ratio (NLR) have been among the most frequently evaluated biomarkers, as each reflects a different component of the inflammatory and immune response to infection [12,13]. In parallel, coagulation-related parameters, particularly the International Normalized Ratio (INR), have also been linked to prognosis through their association with sepsis-induced coagulation abnormalities and systemic disease severity [14,15]. Established clinical instruments provide additional prognostic information. The Sequential Organ Failure Assessment (SOFA) score reflects the extent of acute organ dysfunction [16,17], whereas the Charlson Comorbidity Index (CCI) captures the burden of chronic underlying disease [18]. Nevertheless, when these variables are interpreted separately, they may provide only a partial view of prognosis, because mortality risk in sepsis is shaped by the interaction between systemic inflammation, coagulation dysfunction, organ failure, and pre-existing clinical vulnerability [19].

In this context, the concept of a composite bioscore has gained increasing interest as an integrative approach capable of bringing together biological, clinical, and contextual information into a unified prognostic assessment model [20]. Such an approach may improve risk stratification by combining complementary dimensions of sepsis severity, rather than relying on isolated biomarkers or conventional clinical scores alone [21]. For adult patients with sepsis and septic shock, this type of model is particularly relevant because short-term outcome depends not only on the intensity of the acute inflammatory response, but also on the degree of organ dysfunction and the baseline comorbidity burden.

Based on this premise, in our previous study, we proposed two composite bioscores, BIO-S and BIO-SC, constructed by integrating PCT, NLR, INR, the SOFA score, and the CCI. In the previous monocentric study, conducted on a cohort of 125 surgical patients with sepsis and septic shock, these bioscores demonstrated high prognostic performance for estimating 28-day mortality risk and for stratifying outcome risk, supporting the usefulness of an integrated approach that combines biological biomarkers with clinical severity scores and comorbidity burden [22]. This prognostic orientation of the bioscore model is consistent with the initial objective of BIO-S and BIO-SC, namely, the short-term assessment of mortality risk in adult patients with sepsis and septic shock.

However, the clinical usefulness of any prognostic score depends not only on its initial derivation, but also on the evaluation of its performance in subsequent patient cohorts and in clinically relevant settings [23,24]. Prognostic models developed in single-center populations may be influenced by case-mix characteristics, baseline mortality risk, local patterns of care, and the distribution of comorbidities. Therefore, before such models can be considered for broader clinical use, their discrimination, calibration, and incremental value over established clinical scores should be assessed carefully [25]. This is especially important in sepsis, where clinical heterogeneity and variable baseline mortality can substantially affect the apparent performance of prediction models.

Thus, the main objective of the present study is to evaluate the clinical performance of the previously proposed BIO-S and BIO-SC composite bioscores for 28-day mortality stratification in adults with sepsis and septic shock. The analysis focuses on their ability to discriminate between survivors and non-survivors, to stratify short-term mortality risk, and to provide prognostic information in relation to established clinical scores and individual biological parameters.

By examining BIO-S and BIO-SC in an adult cohort with sepsis and septic shock, the present study aims to clarify their practical value as adjunctive prognostic tools for early mortality risk stratification. This approach may contribute to a more structured interpretation of inflammatory, coagulation, organ dysfunction, and comorbidity-related information in critically ill patients, while supporting a clinically applicable framework for individualized risk assessment.

## 2. Materials and Methods

### 2.1. Study Design and Analyzed Population

The present study was designed as a prospective, observational, monocentric cohort study conducted between January 2022 and December 2024 at “Sfântul Apostol Andrei,” Emergency Clinical County Hospital, Galați, Romania. The study enrolled adult patients admitted to medical and surgical departments with sepsis or septic shock and evaluated the clinical performance of the previously proposed BIO-S and BIO-SC composite bioscores for 28-day mortality stratification.

The analyzed population comprised 572 adult patients with proven or suspected infection associated with sepsis-related organ dysfunction. All patients were evaluated at admission, and their clinical evolution was followed prospectively until discharge, death, or completion of the 28-day follow-up period. Sepsis and septic shock were defined according to the Sepsis-3 consensus definitions [26]. Sepsis was defined as life-threatening organ dysfunction caused by a dysregulated host response to infection, operationalized by an increase in the Sequential Organ Failure Assessment (SOFA) score of at least 2 points from baseline. Septic shock was defined as sepsis associated with persistent circulatory and metabolic abnormalities, requiring vasopressor therapy to maintain a mean arterial pressure of at least 65 mmHg and a serum lactate concentration greater than 2 mmol/L despite adequate fluid resuscitation.

For each patient, demographic data, clinical diagnosis, source of infection, relevant comorbidities, biological parameters, severity scores, length of hospitalization, discharge status, and 28-day survival status were recorded. The primary outcome of interest was 28-day mortality. Patients were subsequently analyzed according to survival status and according to the presence of sepsis or septic shock, in order to assess the prognostic performance of BIO-S and BIO-SC across clinically relevant severity categories.

### 2.2. Inclusion and Exclusion Criteria

Eligible patients were adults aged 18 years or older, admitted to medical or surgical departments with sepsis or septic shock, and presenting with proven or clinically suspected bacterial infection associated with sepsis-related organ dysfunction. Inclusion required the availability of all biological and clinical variables necessary for calculating the BIO-S and BIO-SC bioscores, namely procalcitonin (PCT), neutrophil-to-lymphocyte ratio (NLR), International Normalized Ratio (INR), Sequential Organ Failure Assessment (SOFA) score, and Charlson Comorbidity Index (CCI). Documented 28-day survival status was also required for inclusion in the prognostic analysis.

Informed consent was obtained from all included patients. In cases in which the patient was unable to provide consent because of impaired consciousness or critical illness severity, consent was obtained from the legally authorized representative, according to the approved study protocol and institutional ethical requirements.

Patients were excluded if they had incomplete biological or clinical data required for bioscore calculation, unavailable 28-day outcome status, hematological malignancies, severe immunosuppression, post-transplant status, or ongoing intensive cytostatic therapy. Patients in whom severe viral infections, including coronavirus disease 2019 (COVID-19), represented the main cause of admission were also excluded, in order to reduce confounding related to non-bacterial systemic inflammatory responses. Patients with a hospital stay shorter than 24 h were excluded only when the standardized admission assessment and the required variables for score calculation could not be completed.

### 2.3. Biological Parameters and Clinical Scores Used in the Construction of the Bioscores

For each patient, the variables required for the calculation of the BIO-S and BIO-SC composite bioscores were collected as part of the initial sepsis assessment. These variables included procalcitonin (PCT), neutrophil-to-lymphocyte ratio (NLR), International Normalized Ratio (INR), Sequential Organ Failure Assessment (SOFA) score, and Charlson Comorbidity Index (CCI).

Blood samples for PCT, complete blood count, and coagulation testing were collected at presentation to the Emergency Department, as part of the initial diagnostic and severity assessment. PCT was reported in ng/mL and was determined by electrochemiluminescence immunoassay. NLR was calculated from the automated complete blood count by dividing the absolute neutrophil count by the absolute lymphocyte count. INR was determined by automated coagulometry.

The SOFA score was calculated after admission, using the earliest available complete clinical and laboratory data from the initial assessment and admission record. The standard SOFA components were used, namely respiratory function assessed by the PaO_2_/FiO_2_ ratio, platelet count, serum bilirubin, cardiovascular status, neurological status assessed by the Glasgow Coma Scale, and renal function assessed by serum creatinine or urine output. The CCI was calculated based on pre-existing comorbidities documented in the medical records.

The BIO-S bioscore was calculated by combining the semiquantitative scores assigned to PCT, NLR, and INR with the SOFA score, according to the scoring framework defined in the original model. The BIO-SC bioscore was calculated by adding CCI to BIO-S, thereby incorporating baseline comorbidity burden into the composite prognostic assessment. The same scoring structure as that used in the original BIO-S and BIO-SC model was applied, without modifying the predefined component categories or score construction rules.

In addition to the variables included in the bioscores, the Acute Physiology and Chronic Health Evaluation II (APACHE II) score was recorded as a comparator clinical severity score. The source of infection was also documented in order to characterize the clinical context in which the bioscores were applied.

### 2.4. Clinical Evaluation and Patient Follow-Up

Patients were evaluated clinically at admission and monitored throughout hospitalization according to routine clinical practice. Clinical evolution was assessed daily during the index hospitalization, with particular attention to hemodynamic status, organ dysfunction, complications, discharge status, and survival.

The primary endpoint of the study was all-cause 28-day mortality. Secondary endpoints included in-hospital mortality, discharge outcome, and length of hospitalization. Length of hospitalization was calculated from the date of admission to the date of discharge or death. For patients discharged alive before day 28, survival status at the 28-day endpoint was established from medical records and post-discharge follow-up documentation. All patients discharged alive before day 28 attended the scheduled follow-up visit after discharge, which allowed confirmation of survival status at or beyond the predefined 28-day follow-up point.

Clinical and laboratory data were prospectively recorded in standardized study forms and subsequently centralized in a unified electronic database. Each patient was assigned an anonymous study code before analysis, and no directly identifiable personal data were used in the statistical database. The collected variables were used to evaluate the association between BIO-S, BIO-SC, clinical severity, hospital course, and 28-day survival.

### 2.5. Statistical Analysis

Statistical analyses were performed using IBM SPSS Statistics for Windows, version 26.0 (IBM Corp., Armonk, NY, USA), and R software, version 4.5.0 (R Foundation for Statistical Computing, Vienna, Austria). Descriptive statistics, between-group comparisons, Kaplan–Meier survival analysis, and Cox proportional hazards models were performed in SPSS. Receiver operating characteristic (ROC) curve analysis, pairwise area under the curve (AUC) comparisons using the DeLong test, and bootstrap validation of AUC estimates were performed in R using the pROC package. Data distribution was assessed using the Kolmogorov–Smirnov and Shapiro–Wilk tests, together with visual inspection of histograms and Q-Q plots. Continuous variables were expressed as mean ± standard deviation (SD) for normally distributed data or as median and interquartile range (IQR) for non-normally distributed data. Categorical variables were expressed as absolute frequencies and percentages.

Between-group differences were evaluated using Student’s *t*-test or the Mann–Whitney U-test, as appropriate, while categorical variables were compared using the χ^2^ test or Fisher’s exact test. Comparisons were performed between survivors and non-survivors and, where relevant, between patients with sepsis and those with septic shock.

The prognostic performance of BIO-S and BIO-SC for predicting 28-day mortality was evaluated by receiver operating characteristic (ROC) curve analysis. The area under the curve (AUC), 95% confidence interval (CI), sensitivity, specificity, positive predictive value (PPV), and negative predictive value (NPV) were calculated. ROC-based optimal thresholds were identified using Youden’s index, defined as J = sensitivity + specificity minus 1. The discriminative performance of BIO-S and BIO-SC was compared with that of conventional clinical scores, including Sequential Organ Failure Assessment (SOFA) and Acute Physiology and Chronic Health Evaluation II (APACHE II), using the DeLong test for paired AUC comparisons.

Internal validation of ROC performance was performed by bootstrap resampling with 1000 iterations, in order to estimate optimism-corrected AUCs and corresponding 95% CIs. Calibration of the prognostic models for 28-day mortality was assessed using the Hosmer–Lemeshow goodness-of-fit test, the Brier score, and calibration plots comparing predicted and observed mortality across risk strata. Predicted probabilities used for the Brier score, Hosmer–Lemeshow test, and calibration plots were obtained from logistic regression models including BIO-S or BIO-SC as continuous predictors.

Survival was analyzed using Kaplan–Meier curves according to BIO-S and BIO-SC risk categories, and differences between survival curves were tested using the log-rank method. ROC-derived optimal cut-off values were used for binary discrimination analyses. For risk stratification and Kaplan–Meier survival analyses, patients were grouped according to the predefined BIO-S and BIO-SC score strata used in the original scoring framework.

Cox proportional hazards regression models were used to evaluate the association between prognostic scores and 28-day mortality. Hazard ratios (HRs) and 95% CIs were reported. Because BIO-S includes the SOFA score and BIO-SC includes both SOFA and the Charlson Comorbidity Index (CCI), regression models were constructed separately for individual clinical scores and composite bioscores, in order to avoid structural collinearity between predictors and their embedded components. Additional adjusted models included age, sex, admission department, septic shock status, and infection source, when applicable. The proportional hazards assumption was assessed before the interpretation of the Cox models.

All statistical tests were two-tailed, and statistical significance was set at *p* < 0.05.

## 3. Results

### 3.1. Construction of the Individual BIO-S and BIO-SC Bioscores

#### 3.1.1. Component Scoring and Integration into the Composite Bioscores

For each patient, the biological parameters included in the original BIO-S model, namely procalcitonin (PCT), neutrophil-to-lymphocyte ratio (NLR), and International Normalized Ratio (INR), were converted into semiquantitative component scores according to predefined intervals. Each biological parameter was assigned a score from 0 to +4, reflecting increasing biological severity. These component scores were then combined with the Sequential Organ Failure Assessment (SOFA) score and, for the extended model, with the Charlson Comorbidity Index (CCI), in order to obtain the final BIO-S and BIO-SC values, as summarized in Table 1.

The BIO-S bioscore was calculated as the sum of the PCT score, NLR score, INR score, and SOFA score. Therefore, BIO-S integrates inflammatory response, immune cell imbalance, coagulation status, and acute organ dysfunction. The BIO-SC bioscore was calculated by adding CCI to BIO-S, thereby incorporating the burden of pre-existing comorbidities into the prognostic model. The same component thresholds and score construction rules as those defined in the original BIO-S and BIO-SC framework were applied in the present analysis, without recalibration of the raw scoring structure.

#### 3.1.2. Calculation of the BIO-S and BIO-SC Bioscores

After assignment of the semiquantitative component scores for procalcitonin (PCT), neutrophil-to-lymphocyte ratio (NLR), and International Normalized Ratio (INR), the BIO-S bioscore was calculated for each patient by adding these three biological component scores to the Sequential Organ Failure Assessment (SOFA) score, according to the following formula:

BIO-S = PCT score + NLR score + INR score + SOFA score.

The BIO-SC bioscore was calculated as an extension of BIO-S by adding the Charlson Comorbidity Index (CCI), according to the following formula:

BIO-SC = BIO-S + CCI.

Thus, BIO-S reflected the combined contribution of inflammatory response, immune cell imbalance, coagulation abnormalities, and acute organ dysfunction, whereas BIO-SC additionally incorporated pre-existing comorbidity burden. The final BIO-S and BIO-SC values were calculated individually for all patients and were subsequently used in the analyses of 28-day mortality discrimination, calibration, survival stratification, and Cox proportional hazards models.

#### 3.1.3. Use of BIO-S and BIO-SC Values in Prognostic Analyses

In the present study, BIO-S and BIO-SC were retained as composite prognostic scores and were not interpreted as direct absolute probabilities of death. Higher values of each bioscore were considered to indicate a greater cumulative prognostic burden, reflecting the combined contribution of biological severity, organ dysfunction, and, for BIO-SC, pre-existing comorbidity burden.

The raw BIO-S and BIO-SC values were used as continuous variables in receiver operating characteristic (ROC) curve analysis and Cox proportional hazards regression models. In addition, predefined score strata were used to classify patients into prognostic risk categories for the analysis of observed 28-day mortality and Kaplan–Meier survival curves, while ROC-derived thresholds were used for binary discrimination analyses.

Because the relationship between composite score values and mortality probability may not be strictly linear, the percentage mortality risk derived by linear interpolation was not used as a primary outcome measure. Instead, prognostic performance was evaluated through discrimination, calibration, observed mortality across risk strata, survival analysis, and Cox proportional hazards models.

### 3.2. General Characteristics of the Study Population

The study included 572 adult patients with sepsis or septic shock, admitted between January 2022 and December 2024. Of these, 418 patients (73.1%) were classified as having sepsis, while 154 patients (26.9%) met the criteria for septic shock. All patients were recruited from “Sf. Apostol Andrei,” Emergency Clinical County Hospital, Galați, Romania, and were admitted to medical or surgical departments according to clinical presentation and severity.

The demographic and clinical characteristics of the analyzed cohort are presented in Table 2. The median age of the overall population was 67 years (IQR: 63–74), with similar values in patients with sepsis and those with septic shock, 67 years (IQR: 64–74) versus 68 years (IQR: 62–74), respectively (*p* = 0.867). Sex distribution showed a predominance of male patients, with 348 men (60.8%) and 224 women (39.2%) in the total cohort, without significant differences between the sepsis and septic shock groups (*p* = 0.672).

Regarding the type of admission department, 337 patients (58.9%) were admitted to medical departments and 235 patients (41.1%) to surgical departments. The distribution between medical and surgical departments was similar in patients with sepsis and septic shock (*p* = 0.965). The median length of hospitalization was 15 days (IQR: 11–22) in the overall cohort, with comparable values between patients with sepsis and those with septic shock, 15 days (IQR: 11–22) versus 16 days (IQR: 12–22), respectively (*p* = 0.264).

At discharge, 386 patients (67.5%) were recorded as survivors and 186 patients (32.5%) as non-survivors. Discharge outcome differed significantly between the two clinical severity groups, with 355 survivors at discharge (84.9%) among patients with sepsis and 31 survivors at discharge (20.1%) among patients with septic shock (*p* < 0.001). Regarding the primary endpoint, 392 patients (68.5%) were alive at day 28, whereas 180 patients (31.5%) died within the 28-day follow-up period. Twenty-eight-day mortality was markedly higher in patients with septic shock than in those with sepsis, 77.9% versus 14.4%, respectively (*p* < 0.001).

The source of infection was also analyzed in clinically relevant categories, as shown in Table 3. Respiratory tract infections were the most frequent source in the overall cohort, followed by abdominal, pelvic, or anorectal infections and other or undetermined sources. The distribution of infection sources did not differ significantly between patients with sepsis and those with septic shock (*p* = 0.515).

### 3.3. Biomarkers, Clinical Scores, and Bioscore Values According to 28-Day Survival Status

Table 4 summarizes the distribution of the individual biomarkers and clinical scores in the overall adult cohort and according to 28-day survival status. The analysis showed that most biological parameters and clinical scores included in the BIO-S and BIO-SC models differed significantly between survivors and non-survivors, supporting their prognostic relevance in the assessment of short-term mortality risk.

Procalcitonin (PCT) values were markedly higher in 28-day non-survivors than in survivors, with median values of 12.40 ng/mL (IQR: 6.96–26.46) versus 2.49 ng/mL (IQR: 2.24–2.88), respectively (*p* < 0.001). The neutrophil-to-lymphocyte ratio (NLR) showed the same pattern, with higher values in non-survivors, 16.12 (IQR: 8.09–28.25), compared with survivors, 6.53 (IQR: 4.52–10.13), *p* < 0.001. International Normalized Ratio (INR) was also significantly increased in patients who died within 28 days, 2.38 (IQR: 1.56–3.06), compared with those who survived, 1.55 (IQR: 1.35–1.90), *p* < 0.001.

Among the clinical scores, Sequential Organ Failure Assessment (SOFA) was substantially higher in non-survivors, with a median value of 9 (IQR: 7–11), compared with 4 (IQR: 2–7) in survivors (*p* < 0.001), indicating a strong association between acute organ dysfunction and 28-day mortality. The Charlson Comorbidity Index (CCI) also differed significantly between the two outcome groups, with higher values in non-survivors, 5 (IQR: 4–8), compared with survivors, 3 (IQR: 2–4), *p* < 0.001. By contrast, Acute Physiology and Chronic Health Evaluation II (APACHE II), although numerically higher in non-survivors, did not show a statistically significant difference between groups in this cohort, 9.5 (IQR: 4.75–24) versus 7 (IQR: 4–22), *p* = 0.296.

These findings indicate that the main biological and clinical components incorporated into the BIO-S and BIO-SC bioscores were significantly associated with 28-day outcome, whereas APACHE II, used as a comparator severity score, showed a weaker discriminatory pattern in the present cohort.

The composite bioscores showed a similar prognostic pattern, with significantly higher values in patients who died within 28 days, as presented in Table 5. The median BIO-S value in the overall cohort was 13 (IQR: 8–17). Patients who died within 28 days had markedly higher BIO-S values than survivors, 17 (IQR: 15–20) versus 9 (IQR: 7–13), respectively (*p* < 0.001).

The BIO-SC bioscore, which additionally incorporates comorbidity burden through the Charlson Comorbidity Index (CCI), showed an even wider separation between outcome groups. The median BIO-SC value was 15 (IQR: 11–22) in the overall cohort, 12 (IQR: 10–16) in 28-day survivors, and 23 (IQR: 20–26) in 28-day non-survivors (*p* < 0.001). These results indicate that increasing composite bioscore values were strongly associated with short-term mortality and support the subsequent assessment of BIO-S and BIO-SC through discrimination, calibration, risk stratification, and survival analyses.

### 3.4. Discriminative Performance and Calibration of BIO-S and BIO-SC for 28-Day Mortality

**Figure 1:**
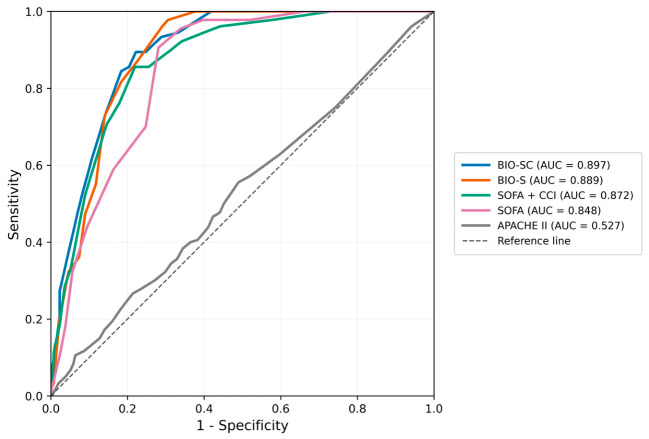
Comparative ROC curves for BIO-S, BIO-SC, SOFA, SOFA plus CCI, and APACHE II in predicting 28-day mortality. The figure shows the discriminatory performance of the composite bioscores and comparator clinical scores for the primary endpoint. BIO-SC had the highest area under the curve (AUC), followed by BIO-S and SOFA plus CCI. The ROC pattern supports the prognostic relevance of integrating biological parameters, organ dysfunction, and comorbidity burden, while APACHE II showed limited discrimination in this cohort.

The discriminative performance of BIO-S and BIO-SC for predicting 28-day mortality was evaluated using receiver operating characteristic (ROC) curve analysis. The results are summarized in Table 6, and the comparative ROC curves are shown in Figure 1. Both composite bioscores showed strong discriminatory ability for 28-day mortality, with an area under the curve (AUC) of 0.889 (95% CI: 0.864–0.915) for BIO-S and 0.897 (95% CI: 0.872–0.921) for BIO-SC.

At the optimal ROC-derived threshold, BIO-S showed very high sensitivity, 97.8%, and a negative predictive value (NPV) of 98.6%, at a cut-off value of 12 points. BIO-SC showed a more balanced profile, with a sensitivity of 89.4%, specificity of 77.8%, positive predictive value (PPV) of 64.9%, and NPV of 94.1%, at a cut-off value of 18 points. Compared with the individual comparator scores, both BIO-S and BIO-SC showed higher AUC values than Sequential Organ Failure Assessment (SOFA) and Acute Physiology and Chronic Health Evaluation II (APACHE II). The combined SOFA plus Charlson Comorbidity Index (CCI) model also showed good discrimination, but its AUC remained lower than that of BIO-SC.

Pairwise AUC comparisons using the DeLong test are presented in Table 7. BIO-SC had a numerically higher AUC than BIO-S, but the difference between the two bioscores was not statistically significant (AUC difference = 0.007, *p* = 0.328). However, BIO-SC significantly outperformed SOFA, SOFA plus CCI, and APACHE II. BIO-S also showed significantly higher discrimination than SOFA and APACHE II, whereas the difference between BIO-S and SOFA plus CCI did not reach statistical significance.

**Figure 2:**
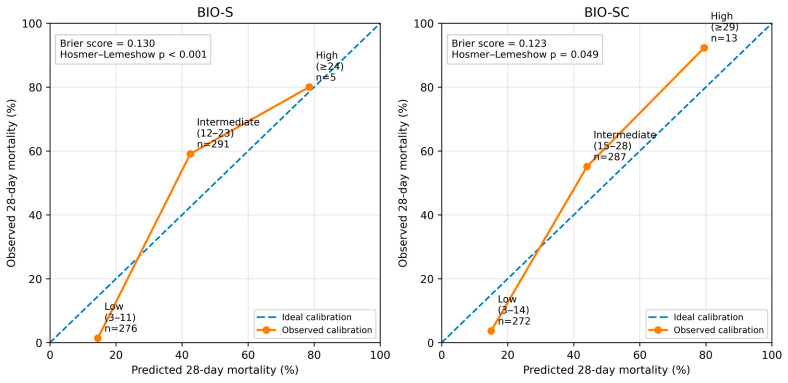
Calibration plots for BIO-S and BIO-SC in predicting 28-day mortality. The plots compare mean predicted probabilities derived from logistic regression models with observed 28-day mortality across predefined risk strata for each bioscore. The diagonal reference line represents perfect calibration, while deviations from this line indicate differences between expected and observed event rates. Both bioscores showed an increasing observed mortality gradient across higher score categories, supporting their use for prognostic stratification rather than direct estimation of individual absolute mortality probability.

Calibration was assessed using predicted probabilities derived from logistic regression models including BIO-S or BIO-SC as continuous predictors. Calibration metrics and observed mortality across predefined risk strata are presented in Table 8, while calibration plots are shown in Figure 2. BIO-SC showed a slightly better calibration profile than BIO-S, with a lower Brier score, 0.123 versus 0.130. The Hosmer–Lemeshow test suggested residual miscalibration for BIO-S and borderline evidence of miscalibration for BIO-SC, indicating that the bioscores should be interpreted primarily as prognostic stratification tools rather than as direct absolute mortality probability models.

Observed mortality increased progressively across predefined score strata. For BIO-S, observed 28-day mortality increased from 1.4% in the low-risk stratum to 59.1% in the intermediate-risk stratum and 80.0% in the high-risk stratum. For BIO-SC, observed mortality increased from 3.7% in the low-risk stratum to 55.1% in the intermediate-risk stratum and 92.3% in the high-risk stratum. These findings support the capacity of both bioscores to stratify mortality risk, with BIO-SC showing a clearer gradient at the upper end of the risk spectrum.

### 3.5. Risk Stratification and Kaplan–Meier Survival Analysis

**Figure 3:**
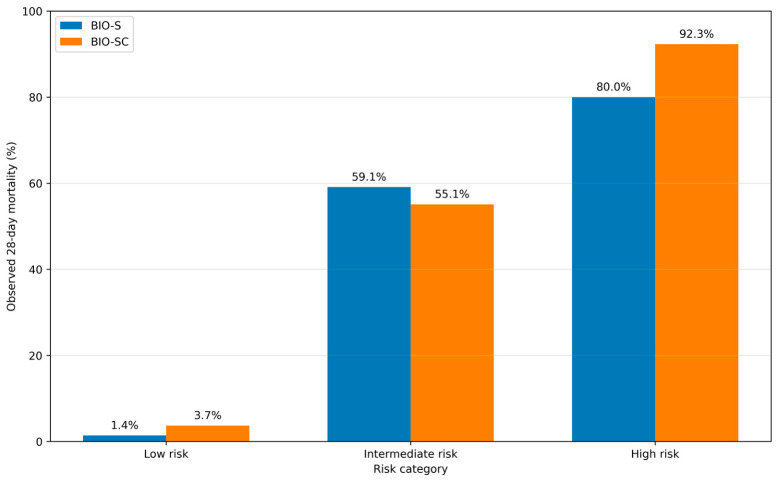
Observed 28-day mortality across BIO-S and BIO-SC risk categories. The figure shows the observed proportion of deaths within 28 days across low, intermediate, and high-risk categories for BIO-S and BIO-SC. Both bioscores demonstrated a progressive increase in observed mortality with increasing risk category, with the highest mortality observed in the high-risk BIO-SC group.

Observed 28-day mortality increased progressively across BIO-S and BIO-SC risk strata, supporting the ability of both bioscores to classify patients into clinically meaningful prognostic categories. For BIO-S, mortality was 1.4% in the low-risk stratum, 59.1% in the intermediate-risk stratum, and 80.0% in the high-risk stratum. For BIO-SC, observed mortality increased from 3.7% in the low-risk stratum to 55.1% in the intermediate-risk stratum and 92.3% in the high-risk stratum. The progressive increase in observed 28-day mortality across the risk categories of both bioscores is illustrated in Figure 3.

Kaplan–Meier survival analysis further confirmed the prognostic separation provided by the two composite bioscores. For BIO-S, 28-day survival remained very high in the low-risk category, 98.6%, but decreased substantially in the intermediate-risk category, 40.9%, and in the high-risk category, 20.0%. The differences between BIO-S risk categories were statistically significant by the log-rank test, χ^2^ = 229.87, *p* < 0.001.

**Figure 4:**
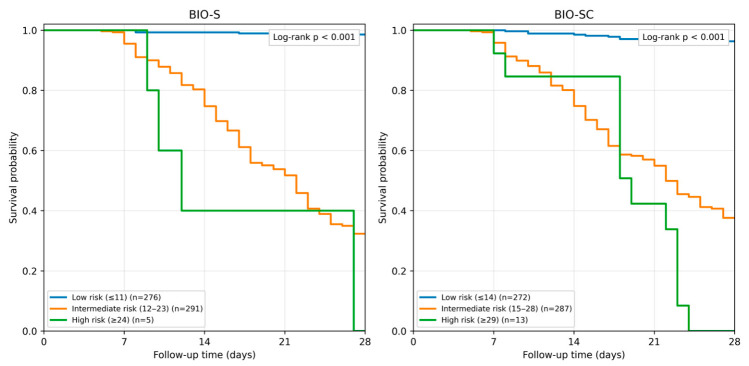
Kaplan–Meier 28-day survival curves according to BIO-S and BIO-SC risk categories. The figure illustrates 28-day survival probability according to low, intermediate, and high-risk categories for BIO-S and BIO-SC. Survival curves showed significant separation between risk categories for both bioscores, with lower survival probabilities observed as bioscore-defined risk increased.

A similar pattern was observed for BIO-SC. Patients in the low-risk category had a 28-day survival probability of 96.3%, whereas survival decreased to 44.9% in the intermediate-risk category and to 7.7% in the high-risk category. The survival curves differed significantly across BIO-SC risk strata, χ^2^ = 195.32, *p* < 0.001. Kaplan–Meier curves for BIO-S and BIO-SC are presented in Figure 4.

These results indicate that both bioscores were able to separate patients according to short-term survival probability. BIO-SC showed a particularly steep decline in survival in the high-risk category, consistent with the additional prognostic contribution of comorbidity burden. However, the highest risk strata, especially for BIO-S, included a limited number of patients, and this should be considered when interpreting the magnitude of survival differences in the upper score ranges.

### 3.6. Cox Proportional Hazards Models for 28-Day Mortality

Cox proportional hazards models were used to evaluate the association between the analyzed biomarkers, clinical scores, composite bioscores, and 28-day mortality. Because BIO-S includes the Sequential Organ Failure Assessment (SOFA) score and BIO-SC includes both SOFA and the Charlson Comorbidity Index (CCI), composite bioscores were not entered in the same multivariable model with their embedded components. Instead, each predictor was evaluated in a separate univariable model and in a separate adjusted model. Adjusted models included age, sex, admission department, septic shock status, and source of infection. Because procalcitonin (PCT) showed a markedly skewed distribution, it was entered into Cox models after natural logarithmic transformation. Because predictors were expressed on different measurement scales, the magnitude of the hazard ratios should not be interpreted as a direct comparison of predictive accuracy between individual biomarkers and composite bioscores.

**Figure 5:**
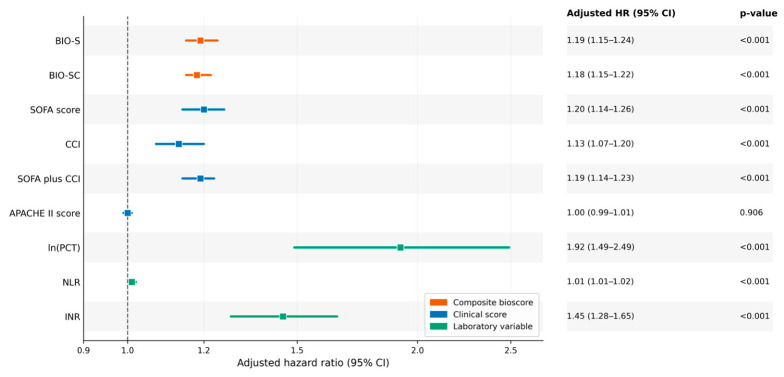
Forest plot of adjusted Cox proportional hazards models for 28-day mortality. The figure illustrates adjusted hazard ratios (HRs) and 95% confidence intervals (CIs) for the analyzed biomarkers, clinical scores, and composite bioscores. Each predictor was evaluated in a separate adjusted Cox proportional hazards model. Adjusted models included age, sex, admission department, septic shock status, and source of infection. Composite bioscores were not entered simultaneously with their embedded components in order to avoid structural collinearity. Procalcitonin (PCT) was entered into the model after natural logarithmic transformation because of its skewed distribution. BIO-S, composite bioscore integrating procalcitonin, neutrophil-to-lymphocyte ratio, International Normalized Ratio, and Sequential Organ Failure Assessment score; BIO-SC, extended composite bioscore integrating BIO-S and Charlson Comorbidity Index; SOFA, Sequential Organ Failure Assessment; CCI, Charlson Comorbidity Index; APACHE II, Acute Physiology and Chronic Health Evaluation II; ln(PCT), natural logarithm of procalcitonin; NLR, neutrophil-to-lymphocyte ratio; INR, International Normalized Ratio.

The Cox regression results are presented in Table 9 and illustrated in Figure 5. In univariable analysis, both composite bioscores were strongly associated with 28-day mortality. Each one-point increase in BIO-S was associated with a higher hazard of death, HR = 1.27, 95% CI: 1.23–1.31, *p* < 0.001. Similarly, each one-point increase in BIO-SC was associated with increased mortality hazard, HR = 1.23, 95% CI: 1.20–1.27, *p* < 0.001.

After adjustment for age, sex, admission department, septic shock status, and source of infection, both bioscores retained a significant association with 28-day mortality. The adjusted HR was 1.19, 95% CI: 1.15–1.24, *p* < 0.001 for BIO-S and 1.18, 95% CI: 1.15–1.22, *p* < 0.001 for BIO-SC. These findings indicate that increasing BIO-S and BIO-SC values remained associated with short-term mortality even after accounting for major clinical and demographic covariates.

Among the comparator scores, SOFA, CCI, and the combined SOFA plus CCI model were also significantly associated with 28-day mortality in both univariable and adjusted analyses. Acute Physiology and Chronic Health Evaluation II (APACHE II) did not show a significant association with 28-day mortality in this cohort, either before or after adjustment. Among the biological variables, ln(PCT), NLR, and INR remained significantly associated with mortality after adjustment, although their prognostic contribution was evaluated separately from the composite bioscores.

## 4. Discussion

The findings of the present study support the prognostic utility of the composite bioscores BIO-S and BIO-SC in adults with sepsis and septic shock and indicate that both models retain strong clinical performance in a larger adult cohort than the original derivation sample. In the previous monocentric study, conducted on adult surgical patients with sepsis and septic shock, BIO-S and BIO-SC showed high performance for 28-day mortality prediction, with BIO-SC presenting the best overall prognostic profile. The present analysis extends this observation to a broader adult cohort, including both medical and surgical admissions, suggesting that the prognostic signal of these bioscores is not limited to the initial surgical population [22].

This continuity between the initial monocentric study and the current adult cohort is important from a methodological perspective. The model was conceived as a prognostic stratification tool, not as a diagnostic instrument, and its central purpose was the assessment of short-term mortality risk. In the present study, BIO-S and BIO-SC were significantly higher in 28-day non-survivors, showed strong discrimination by ROC analysis, separated survival curves across risk categories, and remained associated with mortality in Cox proportional hazards models. These findings support the clinical relevance of the original bioscore concept, while also indicating the need for further independent external validation before broader implementation [22].

Against the background of the existing literature, our results fit into the current tendency to move beyond the isolated use of traditional clinical scores and single biomarkers. Scores such as SOFA and APACHE II remain important benchmarks for the assessment of severity and mortality risk, but they do not fully capture the biological heterogeneity of sepsis. In parallel, individual biomarkers, including PCT and NLR, may provide useful prognostic information, although their isolated performance is generally insufficient for robust risk stratification across heterogeneous septic populations. For this reason, multimarker approaches and composite scores have become increasingly relevant in recent years [27,28,29].

A central element of the present study is that BIO-S and BIO-SC integrate complementary dimensions of sepsis severity. BIO-S combines biological inflammation, immune cell imbalance, coagulation disturbance, and acute organ dysfunction through PCT, NLR, INR, and SOFA. BIO-SC extends this structure by adding CCI, thereby incorporating chronic comorbidity burden into the prognostic assessment. This architecture is clinically plausible because mortality in sepsis is determined not only by the intensity of the acute infectious and inflammatory process but also by the patient’s baseline vulnerability. In our cohort, PCT, NLR, INR, SOFA, and CCI differed significantly between survivors and non-survivors, supporting the biological and clinical rationale for their inclusion in the composite models. This is consistent with studies showing that combining biomarkers with clinical severity scores can improve prognostic assessment in sepsis. For example, Sen and colleagues reported that a bioscore constructed from CRP, PCT, and SOFA had better performance than individual markers for septic classification, although SOFA remained stronger for 28-day prognosis, and the performances were similar at 90 days. By comparison, our results suggest that the additional inclusion of NLR, INR, and comorbidity burden may strengthen short-term mortality stratification [30].

The incremental role of comorbidities remains one of the most relevant aspects of BIO-SC. In the present analysis, BIO-SC showed the highest AUC among the evaluated models and a slightly better Brier score than BIO-S. It also provided a clearer mortality gradient in the highest risk stratum, where observed 28-day mortality reached 92.3%. However, the difference between BIO-SC and BIO-S was not statistically significant in the DeLong comparison, and this finding should temper any claim of definitive superiority. Therefore, BIO-SC should be interpreted as an extended model with numerically stronger performance and better integration of chronic vulnerability, rather than as a statistically superior model to BIO-S in all analyses. This distinction is important for a balanced interpretation of the results and is consistent with the conceptual difference between the two bioscores [22].

The present findings are also aligned with recent studies supporting multimarker strategies in sepsis prognosis. Lee and colleagues showed that a multibiomarker approach based on four biomarkers, evaluated together with SOFA, improved prognostic performance compared with SOFA alone for in-hospital mortality and improved risk reclassification. Their results suggest that integrating several biological axes may provide additional prognostic information beyond conventional severity scores [31]. Our study follows the same conceptual direction, but adds coagulation status and comorbidity burden to the biological and organ dysfunction components, which may explain the strong performance of BIO-S and BIO-SC for 28-day mortality stratification.

Another important aspect is that the value of a composite model does not derive only from adding more variables, but from selecting components with different and complementary pathophysiological meanings. PCT reflects the systemic response to bacterial infection, NLR provides a synthetic measure of inflammatory and immune imbalance, INR captures the coagulation component of sepsis, SOFA quantifies acute organ dysfunction, and CCI expresses pre-existing biological vulnerability. Taken together, these variables describe the septic process more comprehensively than any single marker or score. For this reason, BIO-S and BIO-SC should not be interpreted merely as numerical aggregations, but as structured clinical tools that integrate several dimensions of risk into a single prognostic framework.

The comparison with conventional scores is particularly relevant. SOFA showed good discrimination and remained significantly associated with 28-day mortality, which is expected, given its central role in defining sepsis-related organ dysfunction. However, both BIO-S and BIO-SC showed higher AUC values than SOFA, and BIO-SC significantly outperformed SOFA and SOFA plus CCI in DeLong comparisons. At the same time, BIO-S did not significantly outperform SOFA plus CCI, which suggests that part of its prognostic information overlaps with organ dysfunction and comorbidity-related severity. APACHE II showed limited discrimination and was not significantly associated with 28-day mortality in the present cohort, indicating that its performance may be less stable in this specific population and analytical framework.

Calibration analysis adds an important nuance to the interpretation of the bioscores. Both BIO-S and BIO-SC showed a progressive increase in observed 28-day mortality across predefined risk strata, supporting their usefulness for prognostic stratification. Nevertheless, the Hosmer–Lemeshow test suggested residual miscalibration for BIO-S and borderline evidence of miscalibration for BIO-SC. These findings indicate that the bioscores should be used primarily to stratify patients into risk categories, rather than to provide direct individualized absolute probabilities of death. This is clinically relevant because a score may be useful for identifying patients at higher or lower risk, even when its calibration as an absolute probability model requires further refinement.

Kaplan–Meier analysis reinforced the stratification capacity of the bioscores. Patients in the low-risk categories had very high 28-day survival, whereas survival decreased markedly in the intermediate and high-risk categories. This separation was statistically significant for both BIO-S and BIO-SC. The high-risk groups, especially for BIO-S, included a small number of patients, and the magnitude of mortality estimates in these upper strata should therefore be interpreted with caution. Even so, the consistent gradient across categories supports the clinical utility of the bioscores as risk stratification tools.

Cox proportional hazards models further confirmed the association between the bioscores and 28-day mortality. Importantly, BIO-S and BIO-SC were analyzed in separate models from their embedded components in order to avoid structural collinearity. After adjustment for age, sex, admission department, septic shock status, and source of infection, both bioscores remained significantly associated with mortality. This supports the robustness of their prognostic signal, although the per-point hazard ratios of BIO-S and BIO-SC should not be compared directly without considering their different score ranges and component structures. The significant associations observed for ln(PCT), NLR, and INR also confirm the relevance of the biological components, but their contribution was evaluated separately from the composite bioscores.

Compared with studies evaluating individual biomarkers or restricted patient subgroups, the present results support the advantage of integrative models. Baldirà and colleagues showed that, in patients with sepsis and SOFA ≤ 6, lactate and mid-regional pro-adrenomedullin (MR-proADM) may contribute to 28-day mortality stratification, although individual AUROCs were moderate in certain subgroups [32]. This type of evidence suggests that single biomarkers may be useful in defined contexts, but may not maintain strong performance across heterogeneous populations. Similarly, Song and colleagues reported that combining biomarkers with SOFA may improve the prediction of 28-day mortality, which is conceptually aligned with the present findings [33].

Recent research has also explored other composite indices with prognostic relevance in sepsis. Sarıdaş et al. showed that the CALLY index, based on CRP, albumin, and absolute lymphocyte count, was significantly associated with 30-day mortality in patients with sepsis. Although CALLY captures inflammation, nutritional status, and immune competence, it does not incorporate organ dysfunction, coagulation abnormalities, or comorbidity burden. The present study supports the same general concept, namely that composite models may provide more clinically meaningful prognostic stratification than isolated markers, while extending this approach through the inclusion of organ dysfunction, coagulation, and chronic vulnerability [34].

From a clinical point of view, the main advantage of BIO-S and BIO-SC is feasibility. All components are available in routine hospital practice or can be calculated from standard clinical and laboratory data. This makes the bioscores suitable for integration into electronic medical records or laboratory information systems. BIO-S may be useful when a rapid assessment of acute inflammatory, coagulation, and organ dysfunction burden is needed, whereas BIO-SC may be particularly relevant in older or comorbid patients, in whom chronic vulnerability substantially modifies short-term prognosis. Used appropriately, these scores could support early recognition of high-risk patients, closer monitoring, and more structured communication between emergency, medical, surgical, and intensive care teams.

Several limitations must be acknowledged. First, the study was monocentric and included patients from a single institutional and regional context, which limits external generalizability. Although the cohort was larger and more clinically heterogeneous than the original derivation sample, independent external validation in other hospitals and healthcare systems remains necessary. Second, biomarker assessment was performed at admission, without analysis of the temporal dynamics of PCT, NLR, INR, or SOFA during the first days of hospitalization. Dynamic changes may provide additional prognostic information. Third, the study did not include a detailed analysis of antimicrobial therapy adequacy, source control, or other therapeutic interventions that may influence survival in sepsis. Fourth, calibration results indicate that the bioscores require further refinement before being used as absolute mortality probability models. Fifth, the high-risk strata included relatively small numbers of patients, especially for BIO-S, which may affect the precision of mortality estimates in the upper score ranges. Despite these limitations, the consistency of the discrimination, observed mortality gradients, survival analysis, and Cox models supports the prognostic relevance of BIO-S and BIO-SC in the analyzed adult cohort.

Future research should focus on independent external validation, threshold recalibration, and evaluation in broader adult sepsis populations. International multicenter studies would allow assessment of performance stability across different healthcare systems, case-mix profiles, infection sources, and baseline mortality risks. Further work should also examine whether serial reassessment of the bioscores during the first 24 to 72 h improves prognostic accuracy. Integration into automated clinical information systems could allow real-time score calculation, repeated updating, and early alerting of clinical teams. Extension of the model with additional biomarkers, such as IL-6, soluble urokinase plasminogen activator receptor (suPAR), presepsin, or lactate, may also be explored, although any increase in complexity should be balanced against clinical feasibility.

The present results also support the potential integration of interpretable bioscores into artificial intelligence algorithms and digital decision-support platforms. Recent literature indicates that AI models may improve mortality prediction in sepsis and septic shock, but challenges remain regarding cohort heterogeneity, bias, limited external validation, transparency, and clinical implementation [35]. In addition, artificial intelligence models specifically based on laboratory medicine data, particularly deep learning approaches trained on biomarker panels, hematological indices, coagulation parameters, and routine biochemical profiles, may identify nonlinear prognostic patterns that are difficult to capture through conventional statistical models. When integrated with clinical variables in multimodal decision-support systems, such approaches could further improve early risk stratification in sepsis and septic shock [36,37]. In this context, BIO-S and BIO-SC offer a potential advantage because they are clinically interpretable, based on routinely available variables, and may serve as transparent inputs for explainable AI systems rather than functioning as opaque prediction tools.

## 5. Conclusions

The present monocentric adult cohort study supports the clinical performance of BIO-S and BIO-SC for 28-day mortality stratification in patients with sepsis and septic shock. Both bioscores showed strong discriminatory ability, significant association with 28-day mortality, and clear separation of survival across predefined risk strata.

BIO-SC showed the highest AUC and a slightly better calibration profile, suggesting that the addition of comorbidity burden may improve prognostic characterization. However, the difference between BIO-SC and BIO-S was not statistically significant in the DeLong comparison, so the extended score should be interpreted as clinically more comprehensive rather than definitively superior.

These findings support the use of BIO-S and BIO-SC as adjunctive prognostic stratification tools based on routinely available clinical and laboratory parameters. Further independent multicenter validation and possible recalibration of risk thresholds are needed before broader clinical implementation.

## Figures and Tables

**Table 1:** Scoring framework and construction of the BIO-S and BIO-SC composite bioscores.

Component	Range or Formula	Assigned Score or Contribution
PCT (ng/mL)	<0.5	0
	0.5–1.99	+1
	2–9.99	+2
	10–49.9	+3
	≥50	+4
NLR	1–3.99	0
	4–5.99	+1
	6–8.99	+2
	9–17.99	+3
	>18	+4
INR	<1.2	0
	1.2–1.49	+1
	1.5–1.99	+2
	2–4.99	+3
	≥5	+4
SOFA score	Standard SOFA score	0–24 points
CCI	Standard CCI score	0–37 points
BIO-S	PCT score + NLR score + INR score + SOFA	0–36 points
BIO-SC	BIO-S + CCI	0–73 points

Legend: PCT, procalcitonin; NLR, neutrophil-to-lymphocyte ratio; INR, International Normalized Ratio; SOFA, Sequential Organ Failure Assessment; CCI, Charlson Comorbidity Index. The table summarizes the semiquantitative scoring system used for the biological components of BIO-S and the integration of SOFA and CCI into the final composite bioscores. BIO-S combines the biological component with acute organ dysfunction, whereas BIO-SC extends this structure by adding comorbidity burden.

**Table 2:** Demographic and clinical characteristics of adult patients with sepsis and septic shock.

Parameter	Total (*n* = 572)	Sepsis (*n* = 418)	Septic Shock (*n* = 154)	*p*-Value
Age, years, median (IQR)	67 (63–74)	67 (64–74)	68 (62–74)	0.867
Sex, *n* (%)				0.672
Male	348 (60.8%)	257 (61.5%)	91 (59.1%)	
Female	224 (39.2%)	161 (38.5%)	63 (40.9%)	
Admission department, *n* (%)				0.965
Medical	337 (58.9%)	247 (59.1%)	90 (58.4%)	
Surgical	235 (41.1%)	171 (40.9%)	64 (41.6%)	
Length of hospitalization, days, median (IQR)	15 (11–22)	15 (11–22)	16 (12–22)	0.264
Discharge outcome, *n* (%)				<0.001
Survivors at discharge	386 (67.5%)	355 (84.9%)	31 (20.1%)	
Non-survivors at discharge	186 (32.5%)	63 (15.1%)	123 (79.9%)	
28-day outcome, *n* (%)				<0.001
28-day survivors	392 (68.5%)	358 (85.6%)	34 (22.1%)	
28-day non-survivors	180 (31.5%)	60 (14.4%)	120 (77.9%)	

Legend: Data are presented as median and interquartile range (IQR) for continuous variables and as absolute frequencies and percentages for categorical variables. Comparisons between patients with sepsis and those with septic shock were performed using the Mann–Whitney U-test for continuous variables and the χ^2^ test or Fisher’s exact test for categorical variables, as appropriate. For categorical variables with two or more categories, the *p*-value refers to the overall comparison of the distribution between groups.

**Table 3:** Source of infection in adult patients with sepsis and septic shock.

Parameter	Total (*n* = 572)	Sepsis (*n* = 418)	Septic Shock (*n* = 154)	*p*-Value
Source of infection, *n* (%)				0.515
Respiratory tract	202 (35.3%)	153 (36.6%)	49 (31.8%)	
Abdominal, pelvic, or anorectal	167 (29.2%)	118 (28.2%)	49 (31.8%)	
Other or undetermined	121 (21.2%)	84 (20.1%)	37 (24.0%)	
Urinary tract	63 (11.0%)	47 (11.2%)	16 (10.4%)	
Skin, soft tissue, or surgical wound	12 (2.1%)	11 (2.6%)	1 (0.6%)	
Bloodstream or catheter-related	7 (1.2%)	5 (1.2%)	2 (1.3%)	

Legend: Data are presented as absolute frequencies and percentages. Infection sources were grouped into clinically relevant categories to avoid excessive fragmentation of the cohort. The *p*-value refers to the overall comparison of infection source distribution between patients with sepsis and those with septic shock, using the χ^2^ test or Fisher’s exact test, as appropriate.

**Table 4:** Biomarker and clinical score values according to 28-day survival status.

Parameter	Total (*n* = 572)	28-Day Survivors (*n* = 392)	28-Day Non-Survivors (*n* = 180)	*p*-Value
PCT (ng/mL), median (IQR)	2.83 (2.28–10.87)	2.49 (2.24–2.88)	12.40 (6.96–26.46)	<0.001
NLR, median (IQR)	7.50 (5.29–17.18)	6.53 (4.52–10.13)	16.12 (8.09–28.25)	<0.001
INR, median (IQR)	1.62 (1.39–2.39)	1.55 (1.35–1.90)	2.38 (1.56–3.06)	<0.001
SOFA score, median (IQR)	6 (3–9)	4 (2–7)	9 (7–11)	<0.001
APACHE II score, median (IQR)	8 (4–22)	7 (4–22)	9.5 (4.75–24)	0.296
CCI, median (IQR)	4 (2–5)	3 (2–4)	5 (4–8)	<0.001

Legend: Data are presented as median and interquartile range (IQR). Comparisons between 28-day survivors and non-survivors were performed using the Mann–Whitney U-test. PCT, procalcitonin; NLR, neutrophil-to-lymphocyte ratio; INR, International Normalized Ratio; SOFA, Sequential Organ Failure Assessment; APACHE II, Acute Physiology and Chronic Health Evaluation II; CCI, Charlson Comorbidity Index.

**Table 5:** BIO-S and BIO-SC values according to 28-day survival status.

Parameter	Total (*n* = 572)	28-Day Survivors (*n* = 392)	28-Day Non-Survivors (*n* = 180)	*p*-Value
BIO-S, median (IQR)	13 (8–17)	9 (7–13)	17 (15–20)	<0.001
BIO-SC, median (IQR)	15 (11–22)	12 (10–16)	23 (20–26)	<0.001

Legend: Data are presented as median and interquartile range (IQR). Comparisons between 28-day survivors and non-survivors were performed using the Mann–Whitney U-test. BIO-S, composite bioscore integrating procalcitonin, neutrophil-to-lymphocyte ratio, International Normalized Ratio, and Sequential Organ Failure Assessment score; BIO-SC, extended composite bioscore integrating BIO-S and Charlson Comorbidity Index.

**Table 6:** Discriminative performance of BIO-S, BIO-SC, and comparator clinical scores for 28-day mortality.

Predictor	AUC (95% CI)	Bootstrap AUC (95% CI)	Optimal Cut-Off	Sensitivity (%)	Specificity (%)	PPV (%)	NPV (%)	Youden Index
BIO-S	0.889 (0.864–0.915)	0.890 (0.864–0.915)	12	97.8	69.4	59.5	98.6	0.672
BIO-SC	0.897 (0.872–0.921)	0.897 (0.873–0.920)	18	89.4	77.8	64.9	94.1	0.673
SOFA	0.848 (0.817–0.878)	0.848 (0.815–0.876)	7	90.6	71.9	59.7	94.3	0.625
SOFA plus CCI	0.872 (0.844–0.901)	0.873 (0.843–0.900)	12	85.6	78.1	64.2	92.2	0.636
APACHE II	0.527 (0.476–0.578)	0.527 (0.474–0.581)	8	55.6	51.0	34.2	71.4	0.066

Legend: AUC, area under the receiver operating characteristic curve; CI, confidence interval; PPV, positive predictive value; NPV, negative predictive value; BIO-S, composite bioscore integrating procalcitonin, neutrophil-to-lymphocyte ratio, International Normalized Ratio, and SOFA score; BIO-SC, extended composite bioscore integrating BIO-S and CCI; SOFA, Sequential Organ Failure Assessment; CCI, Charlson Comorbidity Index; APACHE II, Acute Physiology and Chronic Health Evaluation II. Optimal cut-off values were identified using Youden’s index.

**Table 7:** Pairwise comparison of AUC values using the DeLong test.

Comparison	AUC, First Model	AUC, Second Model	AUC Difference	DeLong *p*-Value
BIO-SC versus BIO-S	0.897	0.889	0.007	0.328
BIO-SC versus SOFA	0.897	0.848	0.049	<0.001
BIO-SC versus SOFA plus CCI	0.897	0.872	0.024	<0.001
BIO-SC versus APACHE II	0.897	0.527	0.370	<0.001
BIO-S versus SOFA	0.889	0.848	0.042	<0.001
BIO-S versus SOFA plus CCI	0.889	0.872	0.017	0.124
BIO-S versus APACHE II	0.889	0.527	0.362	<0.001

Legend: Pairwise comparisons were performed using the DeLong test for correlated ROC curves. AUC, area under the receiver operating characteristic curve; BIO-S, composite bioscore; BIO-SC, extended composite bioscore; SOFA, Sequential Organ Failure Assessment; CCI, Charlson Comorbidity Index; APACHE II, Acute Physiology and Chronic Health Evaluation II.

**Table 8:** Calibration metrics and observed 28-day mortality across BIO-S and BIO-SC risk strata.

Bioscore	Risk Stratum	Score Range	Patients, *n*	Mean Predicted 28-Day Mortality (%)	Observed 28-Day Deaths, *n* (%)	Brier Score	Hosmer–Lemeshow *p*-Value
BIO-S	Low	3–11	276	5.6	4 (1.4%)	0.130	<0.001
	Intermediate	12–23	291	54.9	172 (59.1%)		
	High	≥24	5	97.0	4 (80.0%)		
BIO-SC	Low	3–14	272	4.9	10 (3.7%)	0.123	0.049
	Intermediate	15–28	287	53.9	158 (55.1%)		
	High	≥29	13	93.4	12 (92.3%)		

Legend: Predicted probabilities were obtained from logistic regression models including BIO-S or BIO-SC as continuous predictors. Calibration was assessed using the Brier score, the Hosmer–Lemeshow goodness-of-fit test, and the comparison between mean predicted and observed 28-day mortality across predefined risk strata. The *p*-value of the Hosmer–Lemeshow test refers to the overall calibration of each bioscore model. BIO-S, composite bioscore; BIO-SC, extended composite bioscore.

**Table 9:** Cox regression analyses for 28-day mortality.

Predictor	Univariable HR (95% CI)	*p*-Value	Adjusted HR (95% CI)	*p*-Value
BIO-S	1.27 (1.23–1.31)	<0.001	1.19 (1.15–1.24)	<0.001
BIO-SC	1.23 (1.20–1.27)	<0.001	1.18 (1.15–1.22)	<0.001
SOFA score	1.33 (1.28–1.39)	<0.001	1.20 (1.14–1.26)	<0.001
CCI	1.30 (1.23–1.37)	<0.001	1.13 (1.07–1.20)	<0.001
SOFA plus CCI	1.26 (1.23–1.30)	<0.001	1.19 (1.14–1.23)	<0.001
APACHE II score	1.01 (0.99–1.02)	0.307	1.00 (0.99–1.01)	0.906
ln(PCT)	2.63 (2.34–2.95)	<0.001	1.92 (1.49–2.49)	<0.001
NLR	1.03 (1.03–1.04)	<0.001	1.01 (1.01–1.02)	<0.001
INR	1.66 (1.49–1.84)	<0.001	1.45 (1.28–1.65)	<0.001

Legend: Cox regression analyses were performed separately for each predictor. Adjusted models included age, sex, admission department, septic shock status, and source of infection. Composite bioscores were not entered simultaneously with their embedded components in order to avoid structural collinearity. HR, hazard ratio; CI, confidence interval; BIO-S, composite bioscore integrating procalcitonin, neutrophil-to-lymphocyte ratio, International Normalized Ratio, and Sequential Organ Failure Assessment score; BIO-SC, extended composite bioscore integrating BIO-S and Charlson Comorbidity Index; SOFA, Sequential Organ Failure Assessment; CCI, Charlson Comorbidity Index; APACHE II, Acute Physiology and Chronic Health Evaluation II; PCT, procalcitonin; ln(PCT), natural logarithm of procalcitonin; NLR, neutrophil-to-lymphocyte ratio; INR, International Normalized Ratio.

## Data Availability

The data that support the findings of this study are available in this article, and further inquiries can be directed to the corresponding author.
